# Development of late blight resistant potatoes by cisgene stacking

**DOI:** 10.1186/1472-6750-14-50

**Published:** 2014-05-29

**Authors:** Kwang-Ryong Jo, Chol-Jun Kim, Sung-Jin Kim, Tok-Yong Kim, Marjan Bergervoet, Maarten A Jongsma, Richard GF Visser, Evert Jacobsen, Jack H Vossen

**Affiliations:** 1 Wageningen UR Plant Breeding, Wageningen University & Research Centre, P.O. Box 386, 6700 AJ Wageningen, The Netherlands; 2 Graduate School Experimental Plant Sciences, Wageningen University, Wageningen, The Netherlands; 3 Research Institute of Agrobiology, Academy of Agricultural Sciences, Pyongyang, DPR Korea; 4 Plant Research International, Wageningen University and Research Centre, Wageningen, The Netherlands

**Keywords:** Potato, Late blight, Resistance gene, Cisgenesis, Marker-free transformation

## Abstract

**Background:**

*Phytophthora infestans,* causing late blight in potato, remains one of the most devastating pathogens in potato production and late blight resistance is a top priority in potato breeding. The introduction of multiple resistance (*R*) genes with different spectra from crossable species into potato varieties is required. Cisgenesis is a promising approach that introduces native genes from the crops own gene pool using GM technology, thereby retaining favourable characteristics of established varieties.

**Results:**

We pursued a cisgenesis approach to introduce two broad spectrum potato late blight *R* genes, *Rpi-sto1* and *Rpi-vnt1.1* from the crossable species *Solanum stoloniferum* and *Solanum venturii,* respectively, into three different potato varieties. First, single *R* gene-containing transgenic plants were produced for all varieties to be used as references for the resistance levels and spectra to be expected in the respective genetic backgrounds. Next, a construct containing both cisgenic late blight *R* genes (*Rpi-vnt1.1* and *Rpi-sto1*), but lacking the bacterial kanamycin resistance selection marker (*NPTII*) was transformed to the three selected potato varieties using *Agrobacterium*-mediated transformation. Gene transfer events were selected by PCR among regenerated shoots. Through further analyses involving morphological evaluations in the greenhouse, responsiveness to *Avr* genes and late blight resistance in detached leaf assays, the selection was narrowed down to eight independent events. These cisgenic events were selected because they showed broad spectrum late blight resistance due to the activity of both introduced *R* genes. The marker-free transformation was compared to kanamycin resistance assisted transformation in terms of T-DNA and vector backbone integration frequency. Also, differences in regeneration time and genotype dependency were evaluated.

**Conclusions:**

We developed a marker-free transformation pipeline to select potato plants functionally expressing a stack of late blight *R* genes. Marker-free transformation is less genotype dependent and less prone to vector backbone integration as compared to marker-assisted transformation. Thereby, this study provides an important tool for the successful deployment of *R* genes in agriculture and contributes to the production of potentially durable late blight resistant potatoes.

## Background

Genetic disease resistance is an effective tool for sustainable management of late blight, caused by *Phytophthora infestans,* which is economically the most important disease of potato. Breeding at the beginning of the twentieth century concentrated on major dominant late blight resistance (*R*) genes from the Mexican wild species *Solanum demissum* and eleven of these *R* genes were introgressed in potato [1-4]. However, rapid breakdown of resistance in potato varieties containing *S. demissum R1, R2, R3,* and *R10*[3,5] has sparked an increased focus on the introgression of multiple broad spectrum *R* genes in order to impart durability to commercial varieties. It has turned out in various crops and pathosystems that stacking of multiple *R* genes is necessary to provide satisfactory resistance in the field [6]. Although the used *R* genes provide resistance to broad spectra of late blight strains, the predominant agricultural deployment of only one *R* gene can drive the evolution of new virulent strains. In the absence of chemical controls this might even result in the destruction of an entire harvest [7]. Therefore, the use of combinations of *R* genes with different spectra must be pursued to increase durability of resistance and thereby providing food security under no or little fungicide application. *R* gene stacking might be achieved by genetic crossings but the desired variety characteristics will never be fully recovered due to the high level of heterozygosity in potato. Sarpo Mira is an example of a durably late blight resistant potato variety which contains a stack of at least four *R* genes [8,9]. Unfortunately, the variety has not acquired a large market share yet because established varieties are preferred by farmers, processors and consumers.

Addition of stacks of cloned *R* genes [10-17] to existing varieties (resistant or susceptible) through genetic modification (GM) technology is therefore an attractive alternative. Moreover, GM technology circumvents the problem of linkage drag and can speed up the introgression of the *R* gene [18,19]. GM technology has, however, met various types of opposition and a major point of criticism concerns the introduction of “foreign” genes into the food chain and environment. However, within the framework provided by cisgenesis only natural genes from the same or crossable species are used [20,21]. Cisgenes are, therefore, already present in the natural gene pool of the crop plant and cisgenesis only facilitates their introduction into crops. Indeed a majority of a broad panel of European consumers find cisgenic apples safe and not harmfull for the environment [22].

Recently, the transformation of three broad spectrum potato late blight resistance genes (*Rpi-sto1, Rpi-vnt1.1* and *Rpi-blb3*) was described in potato [23]. *Rpi-sto1, Rpi-vnt1.1* and *Rpi-blb3* are native genes from crossable species and are therefore considered as cisgenes for potato. However, the plants in the study from Zhu et al. [23] are “transgenic” as the selectable marker gene, NPTII, was of bacterial origin. Also beyond the cisgenesis framework it is not desired to introduce antibiotic resistance genes into the environment and in this study, we established a pipeline for *Agrobacterium*-mediated transformation of potato in the absence of a selectable marker gene (marker-free transformation). After the absence of vector backbone integration was confirmed, these potatoes were designated as “cisgenic” because of the absence of any foreign (non-potato) genes. This is the first scientific report on the production and functional evaluation of cisgenic *R* gene stacking in different potato varieties.

## Results

### Transformation and functional expression of single late blight *R* genes in potato varieties

**Figure 1:**
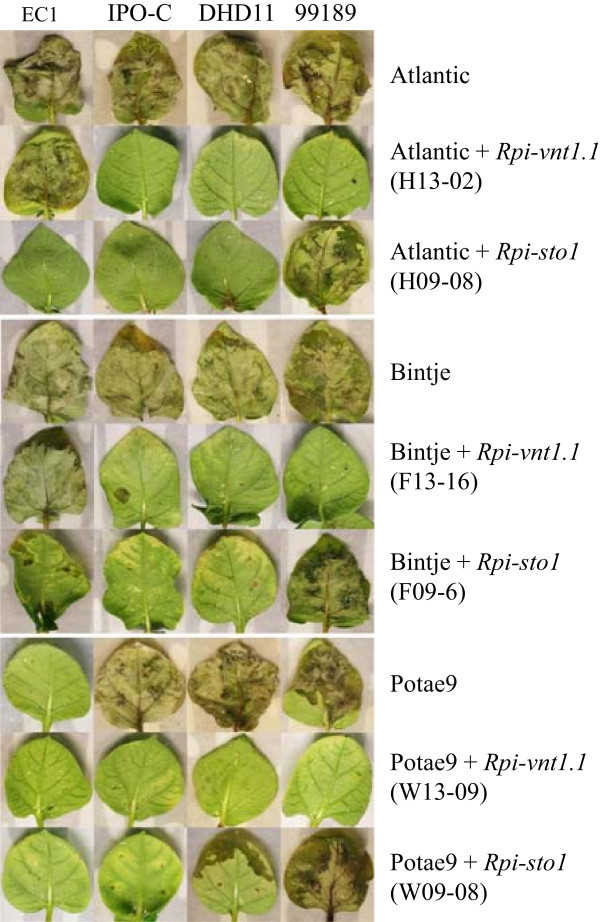
**Detached leaf assays of transgenic potatoes obtained by marker-assisted transformation with single *R *gene constructs.** Non transformed Atlantic or Bintje were susceptible to four *P. infestans* isolates. *Rpi-vnt1.1-*containing transgenic plants were susceptible to EC1 and *Rpi-sto1-*containing transgenic plants were susceptible to pic99189.

The resistance spectra of three potato varieties (the American variety Atlantic, the Dutch variety Bintje and the Korean variety Potae9) were tested with five *P. infestans* isolates with variable virulence spectra and aggressiveness. Atlantic and Bintje were susceptible to all tested isolates while Potae9 was resistant to two isolates (EC1 and 90128; Table 1). These two isolates are a-virulent on plants carrying *R2* type of resistance genes. The presence of *R2* or a functional homolog in Potae9 was confirmed using AVR2 response experiments (data not shown). In order to make Atlantic and Bintje resistant to late blight and to broaden the resistance spectrum of Potae9, these three varieties were transformed with two constructs (pBINPLUS:*Rpi-vnt1.1* and pBINPLUS*:Rpi-sto1* harbouring the kanamycin resistance gene *NPTII*), each containing a single late blight *R* gene . The transgenic events were collected using selection for kanamycin resistance and, successively, the functional expression of the introduced *R* genes was tested using agroinfiltration of the cognate a-virulence (*Avr*) genes. Also the transgenic events were subjected to *P. infestans* inoculation using a detached leaf assay (DLA; Table 1). As an example, the interactions of a representative set of transgenic events with the selected isolates are shown in Figure 1. As expected, the majority of the transgenic events showed resistance to at least four of the five tested *P. infestans* isolates. EC1 and pic99189 were described previously to break the *Rpi-vnt1.1* and *Rpi-sto1* mediated resistances, respectively [13,24]. Indeed, transgenic Atlantic and Bintje events harbouring the *Rpi-vnt1.1* gene were susceptible to isolate EC1. The Potae9 transgenic events containing *Rpi-vnt1.1* were resistant to EC1, due to the presence of *R2* or a functional homolog in Potae9. The *Rpi-sto1-*containing events were susceptible to isolate pic99189. It is concluded that both *Rpi-vnt1.1* and *Rpi-sto1* were able to confer resistance in the selected varieties and these two genes may, therefore, be combined as a cisgenic *R* gene stack in the selected varieties.

**Table 1:** **List of transgenic reference plants obtained by single *R *** **gene transformation**

**Variety**	**Introduced *R *** **gene**	**Plant ID**	**PCR**		**Agroinfiltration**		**DLA**				
			***vnt1.1***	***sto1***	***Avrvnt1***	***Avrsto1***	**EC1**	**IPO-C**	**DHD11**	**90128**	**pic99189**
Atlantic	n	H	**-**	**-**	**-**	**-**	S	S	S	S	S
	*Rpi-vnt1.1*	H13-2	+	**-**	+	**-**	S	R	R	R	R
	*Rpi-sto1*	H9-10	**-**	+	**-**	+	R	R	R	R	S
Bintje	n	F	**-**	**-**	**-**	**-**	S	S	S	S	S
	*Rpi-vnt1.1*	F13-10	+	**-**	+	**-**	S	R	R	R	R
	*Rpi-sto1*	F9-4	**-**	+	**-**	+	R	R	R	R	S
Potae9	n	W	**-**	**-**	**-**	**-**	R	S	S	R	S
	*Rpi-vnt1.1*	W13-8	+	**-**	+	**-**	R	R	R	R	R
	*Rpi-sto1*	W9-1	**-**	+	**-**	+	R	R	R	R	S

*n*, no transformation; - = not detected or not responsive to agroinfiltation; + = PCR positive or responsive to agroinfiltration; R, resistant; S, susceptible; DLA: detached leaf assays with the indicated *P. infestans* isolates.

### Selection and validation of cisgenic potato plants with two late blight *R* genes

**Figure 2:**
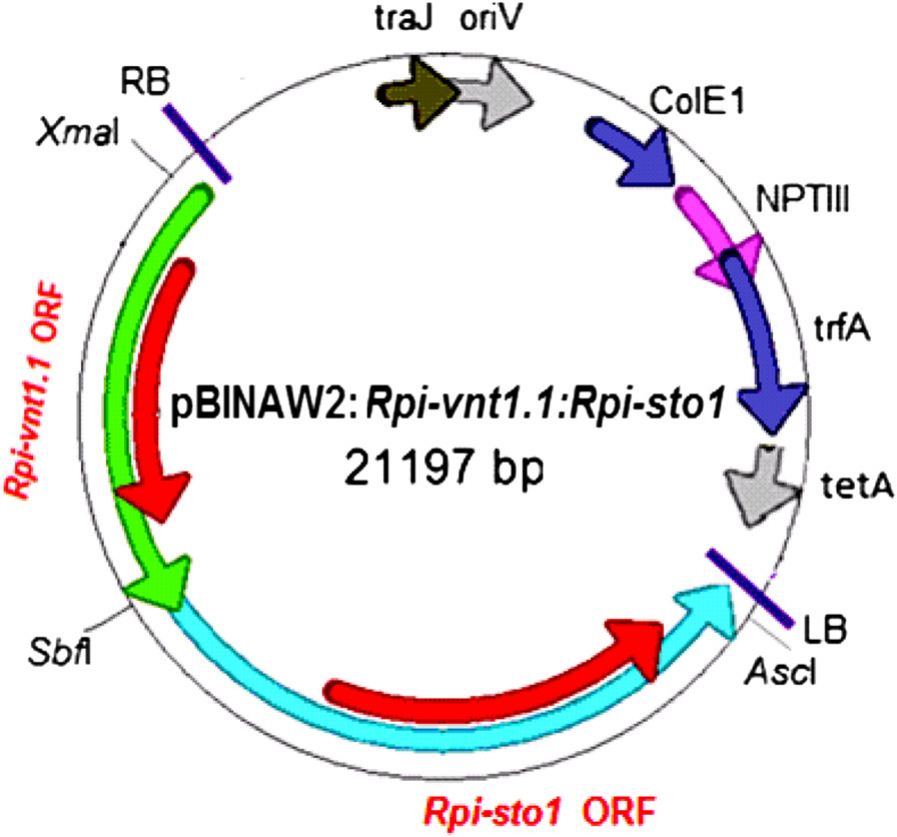
**Schematic diagram of the marker-free double gene construct pBINAW2:*Rpi-vnt1.1*:*Rpi-sto1*.** In light green and light blue arrows the *Rpi-vnt1.1* and *Rpi-sto1* genes are shown, respectively. The red arrows indicate the coding regions of *Rpi-vnt1.1* or *Rpi-sto1*. Unique restriction enzyme recognition sites *Xma*I, *Sbf*I and *Asc*I are shown. RB: right border of T-DNA, LB: left border of T-DNA, TetA, trfA, NPTIII, ColE1, oriV and traJ are vector backbone sequences for plasmid stability and replication in bacterial hosts *Agrobacterium tumefaciens* and *Escherichia coli*.

**Figure 3:**
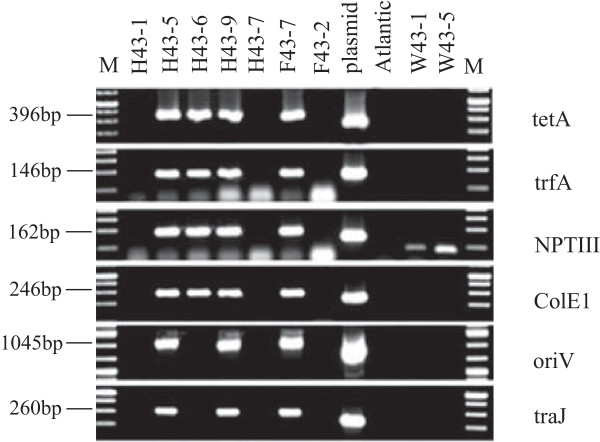
**Vector backbone integration in marker-free transformation events.** Atlantic (H), Bintje (F) and Potae9 (W), were transformed with construct pBINAW2: *Rpi-*v*nt1.1:Rpi*-*sto1*. PCR analysis was performed using primers specific for tetA, trfA, NPTIII, ColE1, oriV and traJ to detect vector backbone integration. The plasmid pBINAW2:*Rpi-vnt1.1:Rpi-sto1* was used as a positive control and the untransformed Atlantic as a negative control. Only the NPTIII primers amplified an a-specific fragment of similar size as shown here for the backbone free events W43-1 and W43-5 in untransformed Potae9. None of the other primers amplified an a-specific band in Potae9 or Bintje (data not shown) M: molecular weight marker.

**Figure 4:**
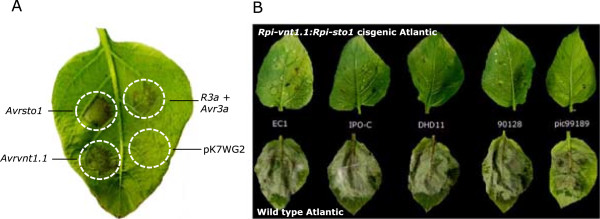
**Functional validation of cisgenic transformants by agroinfiltration and resistance assays. A**. *Avrvnt1-* and *Avrsto1-*induced hypersensitive responses in cisgenic transformant H43-7 (*Rpi-vnt1*:*Rpi-sto1* in Atlantic background). *Avrvnt1 and Avrsto1* were infiltrated in cisgenic plants. A 1:1 mixture of *R3a* and *Avr3a* and pK7WG2 were infiltrated as positive and negative controls, respectively. **B**. Detached leaf assays for cisgenic transformant H43-7. Different isolates are shown in the middle. Cisgenic transformant are shown on the top and the wild type Atlantic on the bottom of the panel.

Cisgenesis excludes antibiotic resistance marker-assisted transformation since the genes encoding the selection markers are derived from non-crossable species. We, therefore, pursued marker-free transformation of the cisgenes *Rpi-vnt1.1* and *Rpi-sto1* in combination with PCR selection (Table 2). Two hundred stem explants from each of the three selected varieties were prepared and co-cultivated with an *A. tumefaciens* strain carrying only the cisgenic late blight *R* genes *Rpi-vnt1.1* and *Rpi-sto1* between the T-DNA borders of a binary plasmid (Figure 2). Between 31 and -110 days after transformation, over 1515 shoots were collected in five rounds of harvesting (Table 3). During the experiment, the shoot regeneration potential of the callus gradually dropped and at 130 days after transformation no more shoots could be harvested. These 1515 shoots were screened by PCR with *Rpi-vnt1* and *Rpi-sto1* primers and 27 PCR positive shoots were selected (Table 2). All PCR positive shoots were originating from different explants, indicating that they were independent transformation events. Two Bintje events only contained the *Rpi-vnt1* gene and were discarded. The remaining 25 events, containing both *Rpi-vnt1* and *Rpi-sto1*, were further tested using vector backbone gene-specific PCR analysis (Figure 3). We found that six events contained vector backbone sequences (Table 2). The remaining 19 events were vector backbone free and are therefore designated as cisgenic events. The 19 cisgenic events were transferred to the greenhouse for phenotypic characterisation. Three weeks after transfer to the greenhouse, five events displayed abnormal plant morphology that consisted of curly leaves and dwarfed growth (Additional file 1), a phenomenon that is commonly observed after regeneration [25]. The five events with these aberrant phenotypes were disregarded for further studies and the remaining 14 events were tested for their responsiveness to *Avrvnt1* and *Avrsto1* after agroinfiltration. Five events responded only to *Avrvnt1* and not to *Avrsto1.* Eight events responded to both *Avrvnt1* and *Avrsto1* infiltration, showing that both *Rpi-vnt1* and *Rpi-sto1* were functionally expressed (Table 4). The latter eight plants also displayed resistance in DLA to all *P. infestans* isolates tested. Figure 4 shows an example of the validation of functional expression for both transferred *R* genes in event H43-7 (Atlantic background) by agroinfiltration and resistance assays in the DLA. Using the single gene-containing transgenic plants as reference it was demonstrated that stacking of *R* genes with different resistance spectra leads to complementary broad spectrum resistance (Table 1). Interestingly, the two introduced *R* genes are complementing the resistance spectrum that was already present in Potae9 plants. Using the pursued experimental setup we were able to select two cisgenic events in Atlantic, five cisgenic events in Bintje and one cisgenic event in Potae9 containing and functionally expressing a stack of two late blight *R* genes.

**Table 2:** **Marker-free transformation of two *R *** **genes (*Rpi-vnt1.1:Rpi-sto1*) to different potato varieties; Marker-free transformation frequencies**

**Variety**	**explants #**	**shoots #**	**PCR + #**	**frequency %**	**bbf #**	**bbf %**
Atlantic	200	497	0/0/12	2.4	9	75
Bintje	200	590	2/0/6	1.0	5	83
Potae9	200	428	0/0/7	1.6	5	71
total	600	1515	2/0/25	1.7	19	76

#explants; number of explants; #shoots: number of shoots tested; #PCR+: the number of shoots containing *Rpi-sto1*, *Rpi-vnt1.1*, or both genes, respectively, as detected by PCR; % frequency: transformation frequency, as percentage of PCR + shoots, carrying both *Rpi-sto1* and *Rpi-vnt1.1*, over the number of tested shoots; #bbf: number of vector backbone free events; % bb: percentage of vector backbone containing PCR + shoots, carrying both *Rpi-sto1* and *Rpi-vnt1.1.*

**Table 3:** **Marker-free transformation of two *R *** **genes (*Rpi-vnt1.1:Rpi-sto1*) to different potato varieties; Identification of PCR-positive shoots in different time ranges after marker-free transformation**

**Variety**	**31-50 days**	**51-70 days**	**71-90 days**	**91-110 days**	**111-130 days**	**Total**
Atlantic	4/197	4/174	4/111	0/15	0/0	12/497
Bintje	2/199	3/194	1/165	0/32	0/0	6/590
Potae9	4/183	2/143	1/78	0/24	0/0	7/428

Number of PCR-positive shoots carrying both *Rpi-sto1* and *Rpi-vnt1.1* over the number of tested shoots.

**Table 4:** **Phenotypic characterization of vector backbone free (cisgenic) events in different potato varieties carrying the *Rpi-vnt1 and Rpi-sto1 *** **genes**

**Cisgenic event**	**Variety**	**Plant morphology**	**Agroinfiltration**		**DLA**				
			***Avrvnt1***	***Avrsto1***	**EC1**	**IPO-C**	**DHD11**	**90128**	**PIC99189**
H43-1	Atlantic		+	–	S	R	R	R	R
H43-2	Atlantic	curly leaf	n	n	n	n	n	n	n
H43-3	Atlantic	curly leaf	n	n	n	n	n	n	n
H43-4	Atlantic		–	–	S	S	S	S	S
**H43-7**	Atlantic		+	+	R	R	R	R	R
**H43-8**	Atlantic		+	+	R	R	R	R	R
H43-10	Atlantic		+	–	S	R	R	R	R
H43-11	Atlantic	curly leaf	n	n	n	n	n	n	n
H43-12	Atlantic		+	–	S	S	S	S	S
F43-1	Bintje		+	–	S	R	R	R	R
**F43-2**	Bintje		+	+	R	R	R	R	R
**F43-3**	Bintje		+	+	R	R	R	R	R
**F43-4**	Bintje		+	+	R	R	R	R	R
**F43-5**	Bintje		+	+	R	R	R	R	R
**W43-1**	Potae9		+	+	R	R	R	R	R
W43-2	Potae9	curly leaf	n	n	n	n	n	n	n
W43-3	Potae9		+	–	S	R	R	R	R
W43-4	Potae9	dwarf	n	n	n	n	n	n	n
**W43-5**	Potae9		+	+	R	R	R	R	R

Cisgenic events functionally expressing both R genes were highlighted by bold font. n: no data, +: responsive to infiltration with the indicated Avr gene; -: not responsive to infiltration with the indicated Avr gene R: resistant to the indicated isolate in detached leaf assays (DLA). S: susceptible to the indicated isolate in DLA.

### Comparison of marker-assisted- and marker-free transformation efficiencies

Kanamycin resistance assisted selection is routinely used for plant transformation. It is, therefore, interesting to compare the efficiency of marker-free transformation in the cisgenesis pipeline to marker-assisted transformation. Marker-assisted transformation efficiency was 100% when expressed as the percentage of rooting shoots being PCR positive for the gene of interest (Table 5). In this definition, marker-free transformation efficiency ranged from 1 to 2.4% over the three varieties.

**Table 5:** **Marker-assisted transformations of single *R *** **genes to different varieties**

**Inserted *R *** **gene**	**Variety**	**Plant ID**	**Explants #**	**Regeneration time (days)**	**shoots %**	**shoots #**	**rooting %**	**PCR + %**	**frequency %**	**vbf %**	**vbf in DLA #**	**vbf and R in DLA #**
*Rpi-vnt1.1*	Atlantic	H13	200	60-120	76	30^a^	100	100	76	40	12	12
*Rpi-sto1*		H09	200	60-120	66	30^a^	100	100	66	50	15	15
*Rpi-vnt1.1*	Bintje	F13	200	60-120	13	26^b^	77	100	10	40	8	8
*Rpi-sto1*		F09	200	60-120	10	20^b^	100	100	10	45	9	9
*Rpi-vnt1.1*	Potae9	W13	200	60-120	16	31^b^	84	100	13	47	12	12
*Rpi-sto1*		W09	200	60-120	19	37^b^	61	100	11	39	9	9

#exp; number of explants, % sht; percentage of number of shoots over number of explants, % rt; percentage of number of rooted shoots over the number of shoots, % PCR+; percentage of PCR positive shoots over the number of shoots, % freq; transformation frequency, calculated by % sht × % rt × % PCR+, a Among regenerated shoots, 30 plants were tested. b All regenerated shoots were tested. % vbf; percentage of backbone free plants out of plants tested. #vbf plants DLA: number of vector backbone free plants tested in detached leaf assays. #vbf R plants DLA: number of vector backbone free resistant plants in detached leaf assay.

For a better comparison of marker-assisted and marker-free transformation, it was essential to use a different definition for transformation efficiency that also takes shoot regeneration efficiency into account. We define marker-assisted transformation frequency as the percentage of PCR positive events among the number of explants used for transformation. Marker-free transformation frequency is defined as the percentage of shoots that is PCR positive. In variety Atlantic a high marker-assisted transformation frequency (71%) was observed whereas the other two varieties, Bintje and Potae9, had significantly lower marker-assisted transformation frequencies (10-13%) (Tables 5 and 6). In marker-free transformation, variety dependent differences in transformation frequencies were less dramatic (2.4, 1.0 and 1.6% in Atlantic, Bintje and Potae-9, respectively) and statistically insignificant (Table 6). Not only the frequency of transformation, also the timing of transformation was different between marker-free and marker-assisted transformation. In the marker-free transformation experiments, the majority of the PCR-positive shoots was obtained between 1 and 3 months after co-cultivation (Table 3). This was quicker than marker-assisted transformation of the *Rpi-vnt1* and *Rpi-sto1* genes individually, which took 2-4 months (Table 5). Finally, we compared vector backbone integration frequencies among the different marker-free and marker-assisted transformation experiments. We did not find significant differences in vector backbone integrations frequency when the different varieties or both of the marker-assisted transformation constructs were compared (Table 7). Only when vector backbone integration frequency was compared between the marker-free (24%; Table 2) and marker-assisted transformation experiments (57%; Table 5) we found that marker-free transformation was associated with less vector backbone integration.

**Table 6:** Pairwise comparisons of transformation frequencies in groups of transformation experiments

**Group 1**			**Group 2**			Pearson analysis*	
	**Non transformed**	**Transformed**		**Non transformed**	**Transformed**	**Chi square**	**P (2-tailed)**
Atlantic (MF)	497	12	Bintje (MF)	590	8	1.6	0.2
Atlantic (MF)	497	12	Potae9 (MF)	428	7	0.7	0.7
Potae9 (MF)	428	7	Bintje (MF)	590	8	0.1	0.7
Atlantic (MA)	15	35	Bintje (MA)	45	5	38	0
Atlantic (MA)	15	35	Potae9 (MA)	44	6	35	0
Potae 9 (MA)	44	6	Bintje (MA)	45	5	0.1	0.7

Transformation frequencies in marker-free transformations (MF) are derived from the number of transformed shoots (transformed) and the total number of shoots minus the number of transformed shoots (non-transformed). Transformation frequencies in marker-assisted transformations (MA) are derived from the number of rooting shoots (transformed) and the number of explants used minus the number of rooting shoots (non-transformed).

*null hypothesis: group 1 equals group 2.

**Table 7:** Pairwise comparisons of vector backbone integration frequencies in groups of transformation experiments

**Group 1**				**Group 2**				Pearson analysis*	
	**Experiments**	**bb**	**bbf**		**Experiments**	**bb**	**bbf**	**Chi square**	**P (2-tailed)**
Atlantic	H09 + H13	28	28	Potae9	W09 + W13	29	20	0.88	0.34
Atlantic	H09 + H13	28	28	Bintje	F09 + F13	22	18	0.23	0.6
Potae9	W09 + W13	29	20	Bintje	F09 + F13	22	18	0.16	0.69
*Rpi-sto1*	H09 + F09 + W09	42	36	*Rpi-vnt1*	H13 + F13 + W13	37	30	0.028	0.86
Markerfree	H43 + F43 + W43	6	21	Marker	H09	13	15	3.560	0.059
Markerfree	H43 + F43 + W43	6	21	Marker	H13	15	13	5.72	0.017

*null hypothesis: group 1 equals group 2; bb: vector backbone containing event; bbf: vector backbone free event. In the experiments columns, H, F, W represent the varieties Atlantic, Bintje and Potae9 respectively. Extensions 09, 13 and 43 represent the constructs pBINPLUS:*Rpi-sto1*, pBINPLUS:*Rpi-vnt1.1*, pBINAW2:*Rpi-vnt1.1:Rpi-sto1*, respectively.

## Discussion

Cisgenesis, is a new approach for traditional plant breeding that uses genetic modification technology to introduce natural genes from within a plant species or from crossable plant species, into varieties [26]. Therefore, any gene “alien” to the breeder’s gene pool can be avoided in the end product which is causal to many environmental and consumers’ concerns about GM food crops [22]. Not only can widely used susceptible varieties, like Bintje and Atlantic, be converted into resistant varieties, also resistant varieties, like Potae9, can be complemented with additional resistance genes to avoid or delay future resistance breakdown. In order to complement existing varieties with stacks of cisgenic *R* genes, two choices must be made: 1. The method to introduce the *R* gene stack and 2. The method to exclude sequences of foreign origin from transformation events. With respect to the introduction method, in this study we chose transformation by marker-free binary vectors and subsequent regeneration in medium without selective antibiotics followed by PCR-based selection of transformation events [27]. Alternatives involving the removal of a selectable marker gene by site specific recombination pose disadvantages because of remnant sequences of foreign origin [28].

The average marker-free transformation frequency was 1.3% and seems to be genotype independent. In a previous marker-free transformation study in potato [27] a T-DNA of 6 kb was transformed with a frequency of 3.5% when *A. tumefaciens* strain AGL0 was used, and 0.4% when *A. tumefaciens* strain LB4404 was used. It can not be concluded that AGL1 + virG, which was used in this study, was less efficient in transferring the T-DNA than AGL0 in the study from de Vetten et al. [27]. From unpublished experiments in our laboratory it is known that regeneration time increases with the size of the T-DNA. We, therefore, assume that the lower transformation frequency in our study is rather related to the larger T-DNA size (11 kb) of the *Rpi-vnt1*:*Rpi-sto1* construct. Therefore, for stacking of more than two genes in cisgenic transformation, the effect of an increased insert size (e.g. >11 kb) on transformation frequency remains to be tested. It is known that marker-assisted transformation frequency is highly genotype dependent in potato [29]. Also here we found that transformation frequencies ranged from 10-71% in different varieties (Table 5). This variation was remarkably less (1-2.4%) in marker-free transformation experiments (Table 2). It must be noted that transformation frequencies can vary between different experiments and that we here only performed a limited number of experiments. However, the currently presented experiments show that marker-free transformation is less prone to varietal differences than marker assisted transformation. This could be caused by differences in antibiotic tolerance between the varieties that provides transformed cells different abilities to develop into a shoot.

In terms of vector backbone integration, marker-free transformation apparently produces a lower percentage (24%) of vector backbone integrations compared to marker-assisted transformation (40-50%). Again, the number of experiments is limited and firm conclusions cannot be drawn. The vector backbone and border sequences in pBINPLUS and pBINAW2 are highly similar and we do not expect that these differences affect vector backbone integration. A potential explanation could be that the presence of the NPTII gene directly next to the left border of the T-DNA would stimulate selection of higher levels of backbone integration. As it is known that left border recognition is inaccurate in *Solanaceae*, [30], especially when agrobacterium strain AGL1 is used [31], positioning of NPTII near the left border would force the integration of the complete T-DNA. So, it might also lead to higher levels of vector backbone integration. In marker-free transformation, six plants out of 14 tested cisgenic plants did not appropriately express *Rpi-sto1* as observed using agroinfiltration of the corresponding *Avr* genes (like H43-1, -10, -12; Table 4). An obvious explanation could be that T-DNA insertion did not proceed all the way to the left border resulting in 3′ truncations of *Rpi-sto1*. These non-functional cisgenic events and the corresponding DNA samples were discarded in an early phase during the selection and, unfortunately, this hypothesis could not be confirmed. We observed and described some cisgenic plants differing morphologically from wild type varieties in the greenhouse (Table 4, Additional file 1). This is a generally observed phenomenon and in tissue culture-based breeding schemes it should be considered that aberrant plant phenotypes must be selected against [29].

According to the established experimental scheme, it takes less than one year to obtain potato plants with cisgenic *R* gene stacks from the *R* gene construct preparation to the functional validation of the resulting cisgenic plants by performing DLAs. As 2-3 shoots per explant can be collected and 30 independent transformed plants are required considering backbone integration and expression, it is recommended that between 1000-1500 explants are to be treated in a marker-free-transformation experiment of potato. The efficiency of PCR analysis can be improved by a factor 10 by pooling ten shoots, so that the labour intensity of the selection of marker-free transformation events is considered reasonable as compared to the marker-assisted transformations. Considering 2-3 years’ field trials, it takes totally 3-4 years to produce late blight resistant cisgenic events in established potato varieties, which can be released for seed tuber multiplication. This time span is remarkably short compared to the conventional breeding scheme. The cisgenic potatoes selected in this study will be further tested for several years to evaluate whether the transferred *R* genes are stably expressed over many vegetative cycles. Chimeras and epigenetic silencing are issues that could affect stability of resistance. Also agronomic performance needs to be assessed and confirmed in multiple growing seasons.

## Conclusions

We have set up and pursued an effective cisgenic marker-free transformation strategy for commercial potato varieties. It was found that marker-free transformation frequency was much less genotype dependent than marker-assisted transformation. Also the frequency of vector backbone integration tended to be lower in the marker-free transformations as compared to the marker-assisted transformations. The susceptibility or the narrow late blight resistance spectra of the selected varieties were upgraded to broad spectrum resistance after the successful introduction of two cisgenic late blight *R* genes. According to the recent conclusion of the European Food Safety Authority GMO Panel, cisgenic plants have a risk level similar to conventionally bred plants [32]. The cisgenic potatoes, generated in this study, will offer a safe, environmentally friendly, alternative to the current agricultural practice which is highly dependent on the use of chemical late blight control agents. For developing countries, where chemical control agents are unaffordable, cisgenic upgrades of local potato varieties might even ensure food security.

## Methods

### Plant material

The potato varieties Atlantic, Desiree, Bintje, and Potae9 were clonally maintained *in vitro* using Murashige and Skoog medium [33] supplemented with 3% (w/v) sucrose at 20°C at Wageningen UR Plant Breeding, Wageningen, The Netherlands. The varieties Potae9 from DPR Korea, which is resistant to late blight, was used for testing its reaction to certain late blight isolates and for transformation experiments to broaden its resistance spectrum.

### *Phytophthora infestans* isolates and late blight resistance tests

Five *P. infestans* isolates (Additional file 2) were used in Detached Leaf Assays (DLAs); The European isolates IPO-C (race 1, 2, 3, 4, 5, 6, 7, 10, 11) and 90128 (race 1, 3, 4, 7, 8, 10, 11); the American isolates, EC1 (race 1, 3, 4, 7, 10, 11) and pic99189 (race 1, 2, 5, 7, 10, 11) and the Korean isolate DHD11 (race 1, 2, 3, 4, 6, 7, 10, 11). The DLAs were performed as described previously [34].

### Vector construction

The single *R* gene constructs used in our study have been described before. Genomic DNA fragments from *S. venturii,* and *S. stoloniferum,* encompassing the *Rpi-vnt1.1* and *Rpi-sto1* genes, respectively, are cloned in the pBINPLUS binary vector [13,16]. The genomic fragments comprise the entire genes including their native promoters and terminators. In order to combine *Rpi-sto1* and *Rpi-vnt1.1* into one markerfree transformation vector, first the *Asc*I and *Sbf*I fragment from the pBINPLUS*:Rpi-sto1* vector, encompassing the *Rpi-sto1* gene, was ligated into the corresponding restriction sites of pBINAW2 [35]. pBINAW2 is a modified version of pBINPLUS where the entire T-DNA, including the NPTII gene, and the adjacent TetR gene from the vector backbone was removed and replaced by a minimal T-DNA containing only left and right border and a small multiple cloning site. To the pBINAW2:*Rpi-sto1* construct, the *Rpi-vnt1.1* gene was added using a *Sbf*I fragment, encompassing the *Rpi-vnt1.1* gene from the pBINPLUS:*Rpi-blb3*:*Rpi-vnt1.1*:*Rpi-sto1* described by Zhu et al. [23]. The clone with the desired *Rpi-vnt1.1* insert orientation, in tandem with *Rpi-sto1* (pBINAW2:*Rpi-vnt1.1*:*Rpi-sto1*, Figure 2; Additional file 3) was selected using restriction analysis. All ligation mixtures was transformed to ElectroMAX *E.coli* DH10b competent cells (Life technologies). Subsequently, the stability of the *R* gene constructs in *Agrobacterium* strain AGL-1 + VirG and functionality of the *R* genes in *N. benthamiana* were carried out using PCR and co-agroinfiltration with corresponding *Avr* genes, respectively. These tests confirmed the stability and activity of the constructs.

### Potato transformation

Marker assisted transformation performed as described previously [36]. Marker-free transformations are derived from this protocol but kanamycin was omitted as a selection agent. Briefly, internodes of 2-5 mm in length were cut from thick stems of 4-week-old *in vitro*-grown plants and were used as explants in transformation experiments. After pre-culture on R3B medium (MS + 3% sucrose + 0.8% agar + 4 mg/ml NAA + 1 mg/ml BAP, pH5.8) supplemented with PACM (MS + 3% sucrose + 0.2% casein hydrolysate + 1 mg/ml 2,4-D + 1 mg/ml kinetin, pH6.5) for two days, explants were inoculated with *Agrobacterium* strain AGL1 + VirG + binary plasmid resuspended in LB medium to an OD_600_ of 0.2. After 2 days cocultivation, the explants were transferred to ZCVK medium (MS + 2% sucrose + 0.8% agar + 1 mg/ml zeatin + 200 mg/ml cefotaxim + 200 mg/ml vancomycin, pH5.8) for regeneration of shoots. Explants were transferred to fresh medium every two weeks. Shoots were transferred to CK medium (MS + 2% sucrose + 0.8% agar + 200 mg/ml cefotaxim + 200 mg/ml vancomycin, pH5.8) to induce root formation. To guarantee that regenerated plants were derived from independent transformation events, only shoots from physically separated positions on each explant were collected. Three weeks later, the rooted plantlets were analysed by PCR to determine the presence of the desired *R* genes. The transformation frequency was calculated as a percentage of the number of *R* gene-PCR positive shoots over the number of tested shoots.

For marker-assisted transformation, 100 mg/ml Kanamycin was added to ZCVK medium and CK medium for selection of transgenic shoots.

### Functional tests of resistance (*R*) genes

Agroinfiltration was performed as previously described [37]. Two leaves per plant from three copies of each of the transformants were infiltrated with the following constructs: two effectors (*Avrvnt1* and *IpiO = Avrsto1*) [13,24], *R3a*[38] and *Avr3a*[39] as the positive control and empty pK7WG2 [40] as the negative control. *Agrobacterium tumefaciens* strain from glycerol stocks was grown in 3 ml of LB medium supplemented with appropriate antibiotics at 28°C overnight. The next day, the cultures were transferred to 15 ml of YEB medium (5 g beef extract, 5 g bacteriological peptone, 5 g sucrose, 1 g yeast extract, 2 ml 1 M MgSO_4_ in 1 litre of milli-Q water) supplemented with antibiotics, 10 μl of 200 mM acetosyringone and 1000 μl of 1 M MES. On the third day, the cells were harvested and resuspended in MMA solution (20 g sucrose, 5 g MS salts and 1.95 g MES in 1 litre of distilled water, adjusted to pH5.6) supplemented with 1 ml of 200 mM acetosyringone to a final OD_600_ of 0.3. Leaves of 4- to 5-weeks old, greenhouse-grown, plants were infiltrated with this suspension. Responses were scored 3 to 4 days after infiltration.

### DNA extraction and polymerase chain reaction (PCR)

Total genomic DNA was isolated from young leaves as described by Fulton et al. [41]. The Retsch machine (RETSCH Inc., Hannover, Germany) was used to grind young plant materials frozen in liquid nitrogen. Primers used for analysis of *R* genes, vector backbone integration are listed in Additional file 4. A pooled sampling method was exploited for PCR analysis of shoots in marker-free transformation. DNA extraction was carried out first by pooling one small leaf from each of ten shoots. If in this first round pools were found which were PCR-positive for both *R* genes and PCR-negative for backbone integration, a second round of PCR was carried out on genomic DNA of individual shoots within the pools. PCR reactions for *Rpi-sto1, Rpi-vnt1.1,* NPTIII, trfA, ColE1, oriV and traJ were performed using DreamTaq^TM^ polymerase (Fermentas) in a standard PCR program (94°C for 60 s followed by 30 cycles of 94°C for 30 s, 58°C for 60 s, 72°C for 90 s and a final extension time of 5 min at 72°C).

### Statistical analysis

Transformation and vector backbone integrations frequencies are binary data and, therefore, the Pearson Chi-square test was chosen to compare the independent samplings of transformation events in the different transformation experiments. Calculations were performed using the IBM SPSS Statistics 22 software pack. Groupwise comparisons with one degree of freedom were applied.

## Competing interests

The authors declare that they have no competing interests.

## Authors’ contributions

JKR wrote the manuscript and was involved in the experimental guidance of KCJ, KTY and MJ established the collaboration between the P-Y and the Wageningen institutes. KTY designed and cloned the marker-free transformation construct. MJ designed the integrated pest management strategy as pursued in the “EuropeAid” and “International Cooperation” projects and he, thereby, provided the scientific blueprint for this study. KCJ, MB and KSJ performed the transformations, PCRs, and late blight resistance assays. RV was involved in manuscript writing. EJ and JV provided the experimental design and supervised the manuscript writing. All authors read and approved the final manuscript.

## Supplementary Material

Additional file 1 Aberrant plant morphologies after transformation and regeneration. Click here for file

Additional file 2 **Characteristics of *P. infestans *** **isolates used in this study.** Click here for file

Additional file 3 **Sequence of the pBINAW2:** ***Rpi-vnt:1.1*:** ***Rpi-sto1 *** **construct.** Click here for file

Additional file 4 Primers used for PCR analysis of transformants. Click here for file
