# Supplementary material for: Development of late blight resistant potatoes by cisgene stacking
Source: BMC Biotechnol. 2014 May 29;14:50. doi: 10.1186/1472-6750-14-50 (PMC4075930; doi:10.1186/1472-6750-14-50)
Supplement: Additional file 1 — Aberrant plant morphologies after transformation and regeneration. [file 1472-6750-14-50-S1.pptx]

## Slide 1
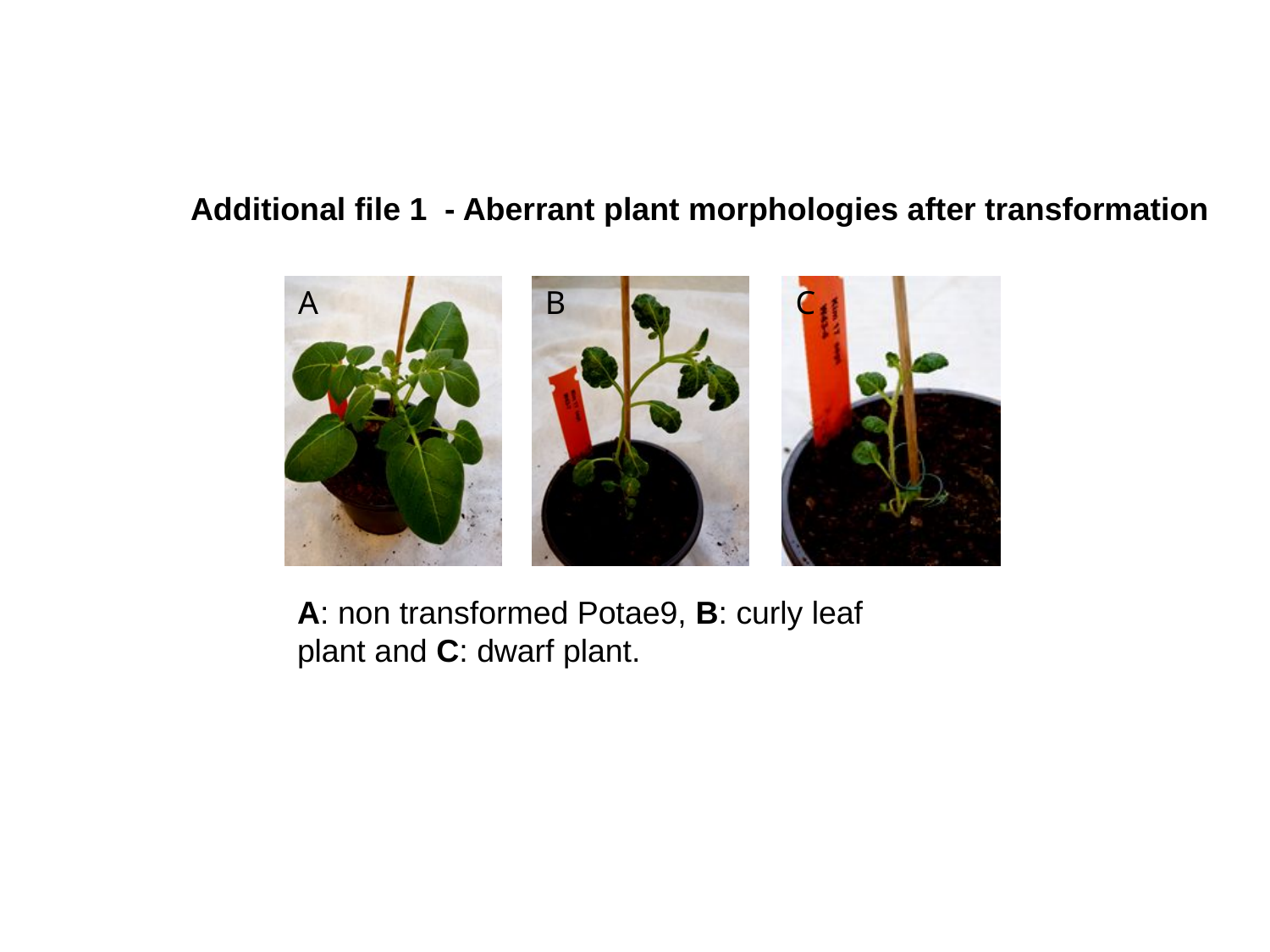

Additional file 1 - Aberrant plant morphologies after transformation
A
C
B
A: non transformed Potae9, B: curly leaf plant and C: dwarf plant.
