# Supplementary material for: Development of late blight resistant potatoes by cisgene stacking
Source: BMC Biotechnol. 2014 May 29;14:50. doi: 10.1186/1472-6750-14-50 (PMC4075930; doi:10.1186/1472-6750-14-50)
Supplement: Additional file 2 — Characteristics of P. infestans isolates used in this study. [file 1472-6750-14-50-S2.pptx]

## Slide 1
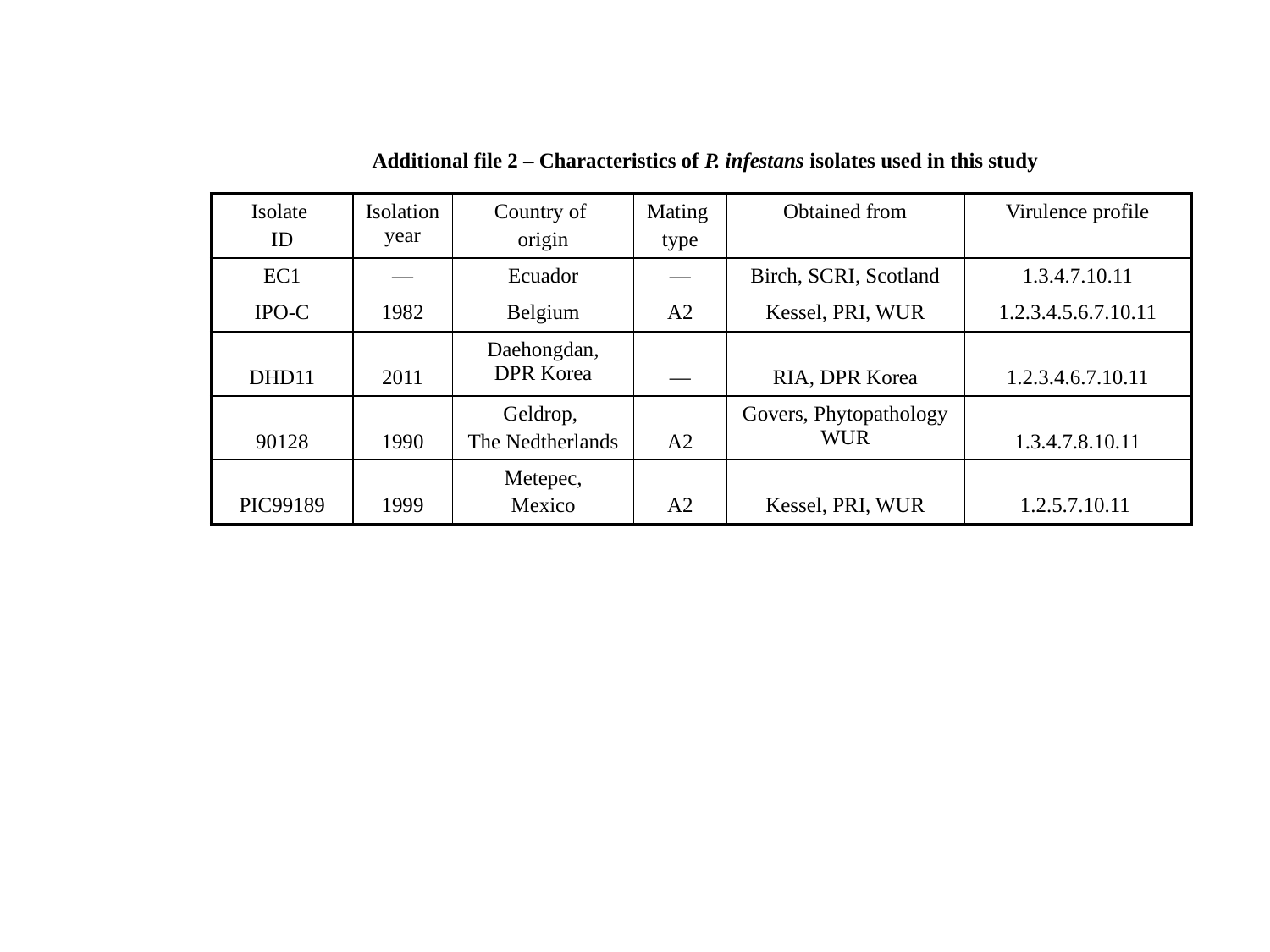

Additional file 2 – Characteristics of P. infestans isolates used in this study
| Isolate ID | Isolation year | Country of origin | Mating type | Obtained from | Virulence profile |
| --- | --- | --- | --- | --- | --- |
| EC1 | — | Ecuador | — | Birch, SCRI, Scotland | 1.3.4.7.10.11 |
| IPO-C | 1982 | Belgium | A2 | Kessel, PRI, WUR | 1.2.3.4.5.6.7.10.11 |
| DHD11 | 2011 | Daehongdan, DPR Korea | — | RIA, DPR Korea | 1.2.3.4.6.7.10.11 |
| 90128 | 1990 | Geldrop, The Nedtherlands | A2 | Govers, Phytopathology WUR | 1.3.4.7.8.10.11 |
| PIC99189 | 1999 | Metepec, Mexico | A2 | Kessel, PRI, WUR | 1.2.5.7.10.11 |
