# Supplementary material for: Development of late blight resistant potatoes by cisgene stacking
Source: BMC Biotechnol. 2014 May 29;14:50. doi: 10.1186/1472-6750-14-50 (PMC4075930; doi:10.1186/1472-6750-14-50)
Supplement: Additional file 3 — Sequence of the pBINAW2: Rpi-vnt:1.1 : Rpi-sto1 construct. [file 1472-6750-14-50-S3.docx]

Additional File 3

>pBINAW2:vnt1:sto1

CTAAGAGAAAAGAGCGTTTATTAGAATAATCGGATATTTAAAAGGGCGTGAAAAGGTTTA

TCCGTTCGTCCATTTGTATGTGCATGCCAACCACAGGGTTCCCCAGATCAGGACCGCTGC

CGGAGCGCAACCCACTCACTACAGCAGAGCCATGTAGACAACATCCCCTCCCCCTTTCCA

CCGCGTCAGACGCCCGTAGCAGCCCGCTACGGGCTTTTTCATGCCCTGCCCTAGCGTCCA

AGCCTCACGGCCGCGCTCGGCCTCTCTGGCGGCCTTCTGGCGCTCTTCCGCTTCCTCGCT

CACTGACTCGCTGCGCTCGGTCGTTCGGCTGCGGCGAGCGGTATCAGCTCACTCAAAGGC

GGTAATACGGTTATCCACAGAATCAGGGGATAACGCAGGAAAGAACATGTGAGCAAAAGG

CCAGCAAAAGGCCAGGAACCGTAAAAAGGCCGCGTTGCTGGCGTTTTTCCATAGGCTCCG

CCCCCCTGACGAGCATCACAAAAATCGACGCTCAAGTCAGAGGTGGCGAAACCCGACAGG

ACTATAAAGATACCAGGCGTTTCCCCCTGGAAGCTCCCTCGTGCGCTCTCCTGTTCCGAC

CCTGCCGCTTACCGGATACCTGTCCGCCTTTCTCCCTTCGGGAAGCGTGGCGCTTTTCCG

CTGCATAACCCTGCTTCGGGGTCATTATAGCGATTTTTTCGGTATATCCATCCTTTTTCG

CACGATATACAGGATTTTGCCAAAGGGTTCGTGTAGACTTTCCTTGGTGTATCCAACGGC

GTCAGCCGGGCAGGATAGGTGAAGTAGGCCCACCCGCGAGCGGGTGTTCCTTCTTCACTG

TCCCTTATTCGCACCTGGCGGTGCTCAACGGGAATCCTGCTCTGCGAGGCTGGCCGGCTA

CCGCCGGCGTAACAGATGAGGGCAAGCGGATGGCTGATGAAACCAAGCCAACCAGGAAGG

GCAGCCCACCTATCAAGGTGTACTGCCTTCCAGACGAACGAAGAGCGATTGAGGAAAAGG

CGGCGGCGGCCGGCATGAGCCTGTCGGCCTACCTGCTGGCCGTCGGCCAGGGCTACAAAA

TCACGGGCGTCGTGGACTATGAGCACGTCCGCGAGCTGGCCCGCATCAATGGCGACCTGG

GCCGCCTGGGCGGCCTGCTGAAACTCTGGCTCACCGACGACCCGCGCACGGCGCGGTTCG

GTGATGCCACGATCCTCGCCCTGCTGGCGAAGATCGAAGAGAAGCAGGACGAGCTTGGCA

AGGTCATGATGGGCGTGGTCCGCCCGAGGGCAGAGCCATGACTTTTTTAGCCGCTAAAAC

GGCCGGGGGGTGCGCGTGATTGCCAAGCACGTCCCCATGCGCTCCATCAAGAAGAGCGAC

TTCGCGGAGCTGGTGAAGTACATCACCGACGAGCAAGGCAAGACCGAGCGCCTTTGCGAC

GCTCACCGGGCTGGTTGCCCTCGCCGCTGGGCTGGCGGCCGTCTATGGCCCTGCAAACGC

GCCAGAAACGCCGTCGAAGCCGTGTGCGAGACACCGCGGCCGCCGGCGTTGTGGATACCT

CGCGGAAAACTTGGCCCTCACTGACAGATGAGGGGCGGACGTTGACACTTGAGGGGCCGA

CTCACCCGGCGCGGCGTTGACAGATGAGGGGCAGGCTCGATTTCGGCCGGCGACGTGGAG

CTGGCCAGCCTCGCAAATCGGCGAAAACGCCTGATTTTACGCGAGTTTCCCACAGATGAT

GTGGACAAGCCTGGGGATAAGTGCCCTGCGGTATTGACACTTGAGGGGCGCGACTACTGA

CAGATGAGGGGCGCGATCCTTGACACTTGAGGGGCAGAGTGCTGACAGATGAGGGGCGCA

CCTATTGACATTTGAGGGGCTGTCCACAGGCAGAAAATCCAGCATTTGCAAGGGTTTCCG

CCCGTTTTTCGGCCACCGCTAACCTGTCTTTTAACCTGCTTTTAAACCAATATTTATAAA

CCTTGTTTTTAACCAGGGCTGCGCCCTGTGCGCGTGACCGCGCACGCCGAAGGGGGGTGC

CCCCCCTTCTCGAACCCTCCCGGCCCGCTAACGCGGGCCTCCCATCCCCCCAGGGGCTGC

GCCCCTCGGCCGCGAACGGCCTCACCCCAAAAATGGCAGCGCTGGCAGTCCTTGCCATTG

CCGGGATCGGGGCAGTAACGGGATGGGCGATCAGCCCGAGCGCGACGCCCGGAAGCATTG

ACGTGCCGCAGGTGCTGGCATCGACATTCAGCGACCAGGTGCCGGGCAGTGAGGGCGGCG

GCCTGGGTGGCGGCCTGCCCTTCACTTCGGCCGTCGGGGCATTCACGGACTTCATGGCGG

GGCCGGCAATTTTTACCTTGGGCATTCTTGGCATAGTGGTCGCGGGTGCCGTGCTCGTGT

TCGGGGGTGCGATAAACCCAGCGAACCATTTGAGGTGATAGGTAAGATTATACCGAGGTA

TGAAAACGAGAATTGGACCTTTACAGAATTACTCTATGAAGCGCCATATTTAAAAAGCTA

CCAAGACGAAGAGGATGAAGAGGATGAGGAGGCAGATTGCCTTGAATATATTGACAATAC

TGATAAGATAATATATCTTTTATATAGAAGATATCGCCGTATGTAAGGATTTCAGGGGGC

AAGGCATAGGCAGCGCGCTTATCAATATATCTATAGAATGGGCAAAGCATAAAAACTTGC

ATGGACTAATGCTTGAAACCCAGGACAATAACCTTATAGCTTGTAAATTCTATCATAATT

GGGTAATGACTCCAACTTATTGATAGTGTTTTATGTTCAGATAATGCCCGATGACTTTGT

CATGCAGCTCCACCGATTTTGAGAACGACAGCGACTTCCGTCCCAGCCGTGCCAGGTGCT

GCCTCAGATTCAGGTTATGCCGCTCAATTCGCTGCGTATATCGCTTGCTGATTACGTGCA

GCTTTCCCTTCAGGCGGGATTCATACAGCGGCCAGCCATCCGTCATCCATATCACCACGT

CAAAGGGTGACAGCAGGCTCATAAGACGCCCCAGCGTCGCCATAGTGCGTTCACCGAATA

CGTGCGCAACAACCGTCTTCCGGAGACTGTCATACGCGTAAAACAGCCAGCGCTGGCGCG

ATTTAGCCCCGACATAGCCCCACTGTTCGTCCATTTCCGCGCAGACGATGACGTCACTGC

CCGGCTGTATGCGCGAGGTTACCGACTGCGGCCTGAGTTTTTTAAGTGACGTAAAATCGT

GTTGAGGCCAACGCCCATAATGCGGGCTGTTGCCCGGCATCCAACGCCATTCATGGCCAT

ATCAATGATTTTCTGGTGCGTACCGGGTTGAGAAGCGGTGTAAGTGAACTGCAGTTGCCA

TGTTTTACGGCAGTGAGAGCAGAGATAGCGCTGATGTCCGGCGGTGCTTTTGCCGTTACG

CACCACCCCGTCAGTAGCTGAACAGGAGGGACAGCTGATAGACACAGAAGCCACTGGAGC

ACCTCAAAAACACCATCATACACTAAATCAGTAAGTTGGCAGCATCACCCATAATTGTGG

TTTCAAAATCGGCTCCGTCGATACTATGTTATACGCCAACTTTGAAAACAACTTTGAAAA

AGCTGTTTTCTGGTATTTAAGGTTTTAGAATGCAAGGAACAGTGAATTGGAGTTCGTCTT

GTTATAATTAGCTTCTTGGGGTATCTTTAAATACTGTAGAAAAGAGGAAGGAAATAATAA

ATGGCTAAAATGAGAATATCACCGGAATTGAAAAAACTGATCGAAAAATACCGCTGCGTA

AAAGATACGGAAGGAATGTCTCCTGCTAAGGTATATAAGCTGGTGGGAGAAAATGAAAAC

CTATATTTAAAAATGACGGACAGCCGGTATAAAGGGACCACCTATGATGTGGAACGGGAA

AAGGACATGATGCTATGGCTGGAAGGAAAGCTGCCTGTTCCAAAGGTCCTGCACTTTGAA

CGGCATGATGGCTGGAGCAATCTGCTCATGAGTGAGGCCGATGGCGTCCTTTGCTCGGAA

GAGTATGAAGATGAACAAAGCCCTGAAAAGATTATCGAGCTGTATGCGGAGTGCATCAGG

CTCTTTCACTCCATCGACATATCGGATTGTCCCTATACGAATAGCTTAGACAGCCGCTTA

GCCGAATTGGATTACTTACTGAATAACGATCTGGCCGATGTGGATTGCGAAAACTGGGAA

GAAGACACTCCATTTAAAGATCCGCGCGAGCTGTATGATTTTTTAAAGACGGAAAAGCCC

GAAGAGGAACTTGTCTTTTCCCACGGCGACCTGGGAGACAGCAACATCTTTGTGAAAGAT

GGCAAAGTAAGTGGCTTTATTGATCTTGGGAGAAGCGGCAGGGCGGACAAGTGGTATGAC

ATTGCCTTCTGCGTCCGGTCGATCAGGGAGGATATCGGGGAAGAACAGTATGTCGAGCTA

TTTTTTGACTTACTGGGGATCAAGCCTGATTGGGAGAAAATAAAATATTATATTTTACTG

GATGAATTGTTTTAGTACCTAGATGTGGCGCAACGATGCCGGCGACAAGCAGGAGCGCAC

CGACTTCTTCCGCATCAAGTGTTTTGGCTCTCAGGCCGAGGCCCACGGCAAGTATTTGGG

CAAGGGGTCGCTGGTATTCGTGCAGGGCAAGATTCGGAATACCAAGTACGAGAAGGACGG

CCAGACGGTCTACGGGACCGACTTCATTGCCGATAAGGTGGATTATCTGGACACCAAGGC

ACCAGGCGGGTCAAATCAGGAATAAGGGCACATTGCCCCGGCGTGAGTCGGGGCAATCCC

GCAAGGAGGGTGAATGAATCGGACGTTTGACCGGAAGGCATACAGGCAAGAACTGATCGA

CGCGGGGTTTTCCGCCGAGGATGCCGAAACCATCGCAAGCCGCACCGTCATGCGTGCGCC

CCGCGAAACCTTCCAGTCCGTCGGCTCGATGGTCCAGCAAGCTACGGCCAAGATCGAGCG

CGACAGCGTGCAACTGGCTCCCCCTGCCCTGCCCGCGCCATCGGCCGCCGTGGAGCGTTC

GCGTCGTCTCGAACAGGAGGCGGCAGGTTTGGCGAAGTCGATGACCATCGACACGCGAGG

AACTATGACGACCAAGAAGCGAAAAACCGCCGGCGAGGACCTGGCAAAACAGGTCAGCGA

GGCCAAGCAGGCCGCGTTGCTGAAACACACGAAGCAGCAGATCAAGGAAATGCAGCTTTC

CTTGTTCGATATTGCGCCGTGGCCGGACACGATGCGAGCGATGCCAAACGACACGGCCCG

CTCTGCCCTGTTCACCACGCGCAACAAGAAAATCCCGCGCGAGGCGCTGCAAAACAAGGT

CATTTTCCACGTCAACAAGGACGTGAAGATCACCTACACCGGCGTCGAGCTGCGGGCCGA

CGATGACGAACTGGTGTGGCAGCAGGTGTTGGAGTACGCGAAGCGCACCCCTATCGGCGA

GCCGATCACCTTCACGTTCTACGAGCTTTGCCAGGACCTGGGCTGGTCGATCAATGGCCG

GTATTACACGAAGGCCGAGGAATGCCTGTCGCGCCTACAGGCGACGGCGATGGGCTTCAC

GTCCGACCGCGTTGGGCACCTGGAATCGGTGTCGCTGCTGCACCGCTTCCGCGTCCTGGA

CCGTGGCAAGAAAACGTCCCGTTGCCAGGTCCTGATCGACGAGGAAATCGTCGTGCTGTT

TGCTGGCGACCACTACACGAAATTCATATGGGAGAAGTACCGCAAGCTGTCGCCGACGGC

CCGACGGATGTTCGACTATTTCAGCTCGCACCGGGAGCCGTACCCGCTCAAGCTGGAAAC

CTTCCGCCTCATGTGCGGATCGGATTCCACCCGCGTGAAGAAGTGGCGCGAGCAGGTCGG

CGAAGCCTGCGAAGAGTTGCGAGGCAGCGGCCTGGTGGAACACGCCTGGGTCAATGATGA

CCTGGTGCATTGCAAACGCTAGGGCCTTGTGGGGTCAGTTCCGGCTGGGGGTTCAGCAGC

CAGCGCTTTACTGGCATTTCAGGAACAAGCGGGCACTGCTCGACGCACTTGCTTCGCTCA

GTATCGCTCGGGACGCACGGCGCGCTCTACGAACTGCCGATAAACAGAGGATTAAAATTG

ACAATTGTGATTAAGGCTCAGATTCGACGGCTTGGAGCGGCCGACGTGCAGGATTTCCGC

GAGATCCGATTGTCGGCCCTGAAGAAAGCTCCAGAGATGTTCGGGTCCGTTTACGAGCAC

GAGGAGAAAAAGCCCATGGAGGCGTTCGCTGAACGGTTGCGAGATGCCGTGGCATTCGGC

GCCTACATCGACGGCGAGATCATTGGGCTGTCGGTCTTCAAACAGGAGGACGGCCCCAAG

GACGCTCACAAGGCGCATCTGTCCGGCGTTTTCGTGGAGCCCGAACAGCGAGGCCGAGGG

GTCGCCGGTATGCTGCTGCGGGCGTTGCCGGCGGGTTTATTGCTCGTGATGATCGTCCGA

CAGATTCCAACGGGAATCTGGTGGATGCGCATCTTCATCCTCGGCGCACTTAATATTTCG

CTATTCTGGAGCTTGTTGTTTATTTCGGTCTACCGCCTGCCGGGCGGGGTCGCGGCGACG

GTAGGCGCTGTGCAGCCGCTGATGGTCGTGTTCATCTCTGCCGCTCTGCTAGGTAGCCCG

ATACGATTGATGGCGGTCCTGGGGGCTATTTGCGGAACTGCGGGCGTGGCGCTGTTGGTG

TTGACACCAAACGCAGCGCTAGATCCTGTCGGCGTCGCAGCGGGCCTGGCGGGGGCGGTT

TCCATGGCGTTCGGAACCGTGCTGACCCGCAAGTGGCAACCTCCCGTGCCTCTGCTCACC

TTTACCGCCTGGCAACTGGCGGCCGGAGGACTTCTGCTCGTTCCAGTAGCTTTAGTGTTT

GATCCGCCAATCCCGATGCCTACAGGAACCAATGTTCTCGGCCTGGCGTGGCTCGGCCTG

ATCGGAGCGGGTTTAACCTACTTCCTTTGGTTCCGGGGGATCTCGCGACTCGAACCTACAGTTGTTTCCTTACTGGGCTTTCTCAGCCCCAGATCTGGGG

TCGATCAGCCGGGGATGCATCAGGCCGACAGTCGGAACTTCGGGTCCCCGACCTGTACCA

TTCGGTGAGCAATGGATAGGGGAGTTGATATCGTCAACGTTCACTTCTAAAGAAATAGCG

CCACTCAGCTTCCTCAGCGGCTTTATCCAGCGATTTCCTATTATGTCGGCATAGTTCTCA

AGATCGACAGCCTGTCACGGTTAAGCGAGAAATGAATAAGAAGGCTGATAATTCGGATCT

CTGCGAGGGAGATGATATTTGATCACAGGCAGCAACGCTCTGTCATCGTTACAATCAACA

TGCTACCCTCCGCGAGATCATCCGTGTTTCAAACCCGGCAGCTTAGTTGCCGTTCTTCCG

AATAGCATCGGTAACATGAGCAAAGTCTGCCGCCTTACAACGGCTCTCCCGCTGACGCCG

TCCCGGACTGATGGGCTGCCTGTATCGAGTGGTGATTTTGTGCCGAGCTGCCGGTCGGGG

AGCTGTTGGCTGGCTGGTGGCAGGATATATTGTGGTGTAAACTCTAGAGGCGCGCCTAGA

GAATGACAGAGAATCGAAAAGCAGCAACGACTGCAATTGGTATCAATTACTCTCAGTGTC

ATATTGTATAATTTTATAAAATTTGTTTAATTTTATACAAGTATCTACTTGTGCACATTC

AGAGTTAAAGGTCATGACTATCCGCTGAAACCAAGTTAAAAATCATGTTTATATATACTA

AAAAAGCATGAATACTTCTATTTGGTGTTTTTTCCACGTGACACAACATTTATTCAGAAA

GTGATAATCACCAACATTATTGTTCGCCAATAATTACAACATTAACAAGTACTCCCTCTG

TCTCAATTTATGTGACACTTTTCATTTTTCGAGAGTCTAGTAATTTAAGTTTGATCGGGA

ATATGCGCATGAAATCTTCTTTTTTTTAAAAAATTAATTTTATATATTTGTAAACTACAT

AAAAAATACTAAAATCTCAATAATTGATAATTCAAAATGTTTAAAAGATCTATGAAAAAT

TTAAGATCAAAGAATCTCGAAATCCAAAAAGTGTCACATAAAATGAGACGAAGGGAGTAT

AATACAGATGGATCTTATTGATTTGTTTTCAAAATAAAATCAGAAAATTCCATACAATAA

GATACTCACAATTTACAAACAAAGAAACAATTACAAATAAGTAATAGATTCTGAAAGCGT

ATAATACAAATAGATTGTTAATTTGTTACCAAAAGAGAATTACATCCAATGAGAGAGAGA

CTCAACAAACAAACAACCCTAGAAAAAAATTACATGCAATCACAATGGCAGGAACCAAAA

AGACTCACAAACAAAGAAACAATAGCAAATAACTTAAATATATATATTCACATTAGGAAT

GTGAGAAATTTTGTGCCAGTCTTCTCCTATTCCCTTCTCACACCGCTTGATCAGTTGTGG

ACATCCCCGAATTTTTAAACTTGTGAGGGTTGTTAGGTGCTGCAATCCCTCTGGTAAACA

TTTTAGCATGTTACAGTGTTCAACAAATAACTCTGTGAGTGAAGATAAACCTTCCAGCCC

TTCCTCAGGGAGACTCTCTAGTGCGCAACACAATTGAATTTTTAGACTTTTCAAAGCATT

CAGACTAGCCAAGCTGGTAGGCAGCTCTTTGAGATTATTGCACCGAGAGATTGTCAAGTA

TTTGAGATTTGCAAGGTTTTTGAACATCTCTTCTGGGAATGAAGTAGCTACTTTATTATA

GCAAATTCTGAGGGAAGTAAGAGCCCTAAGATTAGAAGAAAGGGTCAGAAAAGGGCACTC

GTGAATTATCATCTCTTCAAGCACAGGGAATTGCTCTTCTCCTTCCTTTTTCAGCAATCC

TTTCAGACTACCAAAGTCCCATATATCAAGTTTCCTCAAGGATGGAAACCTTATTCTTGT

GGGGAATCCAGAATGAACATCAATATCCACTTCTTCAACATACTCCACATCCGCAGACCC

CCAGTGTAACTCTAGACTTTCTAGACAAGGCAGATCACCAAAGGGTGGTAAGCATGAGCA

GTTTCTGAAGTTGCTAATTAGAATAGAGACAATATTTTTCAATACTGAGTGATTCATCCA

CTCTGGGAGATGGATTCCTCTGAAGCCATAGATTTTTAAAGAAGTCAGATTGGAGTGTGG

TTTGAGGGCTTCAAGCACTTTAACTTCTTCTGATTCATATATATGTGGTCCAAAGTTATT

CCAACTCATGCTTAAAGAATGCAGATTCCCTTTTGCAGATAAATTGGCTTCTTTTGCGTC

CTTATCATTCTTCACTCTCTCAAGATGCGAGATTTTAATTGAGCCATAGAGATTTAGGTT

TCCTAGTTCACCAAGTTGATAACCTTTCTTCCTTCCAACAACAAATTGACCTAGAGTCTT

AAGGCATGTCAATGATCCTATCCTTGGTGGCATACAAGTCAATGACTGGCTACCATCAAG

TAAAAGATTTCGGAGACTACCAAGTTTACTTGTTTCTTTTGGCAAACAACAAAGCTTGGT

GCAATATTGTAGATCAAGAGTTTGCAGATTTTGAAGCTTGCATAACTGCTTTGGAAGACT

ACGCATGCCACTGCCATACAGGTTCAAGTATCTTAAATGTACTAGATCTCCAATGGAAGA

TGGTAACTTATTAAATGTCGAATCACCTAGATTAAGCACTCTTAACGAGATAAACTTTTC

CAAGGGGGGAAGAGTGTAAAAAAACACCACTTCGGCGAAACCAATGGACATCATATGTGT

GTAACTGTGTTTATTTATTTCACGGATATTGCTGCTTGATGTGTTTGCTGAAAACAGAGA

TGTTGCCAAATCATGGATGAGATCATGCATCTTGAAATAAGTTTTACCATCTTTAACTTC

AATCTCTTGGAAAAAAGACCTCAAGTATAATTCTTTCCATACTTCATCGCCCACATCCTC

TAGCTCCATGTTTCCTTTTGATAAAAGAAAACCATGCGCCATCCAGAGAGAGATTAGCTT

TTCTTTTTCCATTTTGGCATCCTTTGGGAACACCGCACAATACGCAAAGCATTGTTTCAA

ATCAAGTGGAAGTTGATGGTAACTAAGCCTCAGGGCAGGCAGAATAGAACTTTCATCTTG

AGGCAAATTCCAAATCGGACTGTCTCTCACATGTTCCCATGCTCTTTCTTCTCTCTTGAA

GCACAAAATACCTCCAAGAGTTTTGGCTGCTAGAGGCACACCACCACTTTTTTTCACAAT

CTCCTTTCCGATTGCCACAAGGTTTGGATTTATTTCTTCTTGGTGTCCAAATGCACGTTG

CATGAACAACAACCAACAATCTTCTTGAGACAGATTTGACAGTTCATATGGTTGCAATGT

TCCCATAATTGATCCAACCTTTTCAAGACGAGTAGTGGTTAGAACAGAAGCACCACTTGC

TCCAACCTTCAAGACTGCTCTTAAATTAGCCCACTTCTGTTGATCTTCATTCCAAACATC

ATCTAAGACAAGCAAGTATCTTTTTCCATTCAGCAACTCCTGAAGCTTCTTTTGAAGTGG

AGCCAAGTCCATCTCACCAAGTAGTGGCCTTCCTTCAATAGATTCTACAATTGCCTTTAT

TAACCTCTTCTCATCAAAATCTTCCGAGACACAAATCCATATTTTGGAATGGAAATGCTC

AGTAACTCTCTGGTCATTGAAGACCATTTGGGCAAGAGTCGTTTTTCCTAATCCCCCCAT

ACCAAGTATTGGGAGGACTGAAAGGTGTTGGGCATCACTAACATTGTTTATTAGGATTTT

CACTATCTCATCTTTCTCTTTGTCTCTTCCATAAACCTGCGGTTCGGTTAATACAGAACC

TGGTGCAAGCAATGTCCACATGAGAATATTATTACATTCAAACTCATTAGGAGACAACAG

AAAATAGAGGATATTTTCGCACAATATACTCCTGTTCTATCAGAAATACTTTAAATATAT

ACCCATTCTACTTTTGGTCCAATATATCCCCTATGTTCCACTCTGAAAGAAATCCAGCCT

AATCAATGACAAGGAAAAACATGTCTAATCTTTTTTTTTCTTTGTTAGACAAAATTTACA

GTCAGCTGTCAGGAAAGGCTACCGTCGTTGGGATTAACATGCCCTGTTTTTAATGGGATT

AAGGACCCAAGTGCAGCACACAGAGAAACAGAATAGAAGAAAACCTCTTTTCTCTGAGCT

GCTTTGTTAAGAAAAGCAAAACGGATCGAGTGTTCCAAGTTTATAGGTAAAGAAAAGAAT

TCAAAGCAAAGGGAATGGGTAGTGGATGAGTTAGACAGGTGGATTTCTAAAGATATCGAG

TATTTAAAAGTTGGGGTGGATAATTTGATTCTGAGCTTGCCAGATTTGACAAGTATATTC

AGATCGAAAGTACAACGAGAGGTATATATACACTATTTACGATAGGACATGAGTATATTT

AACCCTTTTCTGAATTTGATAATTGCCAAAAAAATGAATATAAACTTAGTTGTTGTAATA

CTAATTTAAGATGAGTACCTGTTTCCCGTCTAACAGCTTGTCTCTCTACAATTTTTTCGT

GCAAATGAAAATTCTTTCTTTCCTCAGCAATTGCCTTTAGTTTTTTCATCACTTGGTCCA

TCCTTTTCCCGACCTTGTGACGGAAAGGGATAACCTTTGGATGATAACGGCCATATTCAG

ACTGGGAGAATCTTGTGGCCTTGGTTTTATATTCATCCAAGATGTCATCGACTTCATATG

TAGCAGCATTGAGTTTTTGCAACCAATTTTCTAGAGGCTTGTTGTTGAGTTGCTTCTCCT

GAGCATCTTCAAGGACGGCTTGAATTGTAGAAAACATGCTTGAAAGCCTTTGGAACTCAT

CTTGAAAACCGAAAAGCAATACAAGTTCCCCTTTGAGGAAAGAAGTGAGATTGTCTAGCA

GAACTTGAATGAAAGCTTCAGCCATATGTGGAAATCACTATTAAGAATATTAGATCTGTG

AATGAATGAATGTGTAATTTTGAAACCAAGATGGCTAGTTGGAATGAAATGGAAAGTTTG

ACTTGTAATAATACAAATTCAATTATTTCATGGAAATTTCACATATGGAATCTCAAAATC

CAAGGCGTTTTGGACCCTTTTAATCTCTCAAGTTGTCAACTTGTTCTTTTGTTTTGTTTT

CTACTCTCTTCATTCATTTTTATTTATCATTGTTTGACCGGACAAATTCATTACGAAAAC

AATTATTCATATAAATATTTTACAAAATAATCCCTATTAAATTGTTTAATACTAAGGATA

AAGCATGAAAAAAATAATTATCTTTTTACATGATAGGTTAAAAGTGATAAATAAAAATAA

AAATCGAAAAGAGTGAGTATTATCTTTTTTTCGTCGACAGCAACTACGCCCCTACTCTTC

CTCATTGGCAGTTTCCTCTTTCCTAATTAATTAAAGCTGAAATAAGTCATTGGCGGATAA

AATGCATAATATAGTGTAGTGAATGGTTAGAATATCACATATCTGTCCGAAAATATTTTA

GAATATATTTTGTCCGTCTCATATTAGATCAGCAGTTTGTATTTTATATATTATTAAGTG

ATGGTCAATAAATTGACAGCTTTTATTATTTTATCCTTTGTTTATATTAATAAGTCTCTT

GCAAAAGAAATTTTGATTCTTCAAAATTTGTTTAAAACTTTTAAAACTATATTAATTGTA

GGGGTAAAATGAGAAAAAATTAATTAATTCTTTTTGATTTAATAAATAATCAAGTAATAT

GAGACAAATATTTTTAGTAAGATGCCCATCTAATATGAGACAGAAGTAGAAAATGAAGAG

AACTGCTAATGGCCCTTGGACCAAAAATGGCCACAAATCTGGAAAGACAATACTTCCTCC

GTCCACTATTAATTATTATACTTTTCATGATTTGAGTCAAATTATAAAAATTTTGACTAA

TATTTTACGATATATTTTTTTATCATATTGATATGCAAAAAATTGCAATGTATAATATTT

TTTGTATAGTTTTTGAATATCTAATTTTTCTGTTTAAAATATTGAATTAGTGTAATCTAA

TTTAGCTTTAAAAATTAGTCAAATTGACTTTCGAAAAGTGCAACATGACAAATCAAAGTG

AACGGATGGAGTACTAATTTTTTGCCTCTAAAATAAAGTTACTCTCTCCATTTCATATTA

ATTGAATTTTTGAGATTTTTTTCATTGTTCAAAATAATTGAATTGTTCAAAGTTTAAGAT

GAATATTTAGTTTCTTTTCCATCTTTGCCCTTTTCTTTGGTGAAATTTGCTTCTAGGAGA

TAAATTGCACTATTTACAAAGTTATATTTATGATTTCACAAAAAACAATAGTGAAAAATA

TGAGTTAAATTATGTCCTTAAATATTTTTCTTAATAAGTTTGTATTACCTCACAAATTTA

ATTAATATAGAAAATTTAATTAATATAGAATAGATGAAGTACTGTTTTCAAAACTCTAAA

AGTAAGTGCAATGAAATTTTTTATGCTAAATGAATTACAATTATCATTAATTAGAAAAAT

TTTAGTAGTTTAGTTGGTTGATTACTTGAACTCTCAACTTGTTGATAAAGTTCGATTCCC

TACTTTGTAATTACCGCCCCATTTTCCTTTTCTCTATACGCAATTAGTAAAAAAAAAAAA

AAAAAAAAAAATTTATCATTAGTATGAAAGGATTATTTAAGAGAATTTTTTTTTATGTCA

CATGGACCCACCACCACGTCATTTCACTTCATCATCTTCCTCTTTTCTCTATTTTTTCAT

CAAATTTTAAATTTAATTTTATTATATATAAATCGTAATTCTTTAAGATTATTTTTGGAC

ATTGTTGTCATCTAATTTCTTTCTTTTTTTTTCATCATATAAAGGCCATTGTATTATAAA

AATTATCATATAATTTTATTATTCTTTTTTATTAAAAAGTCGTTCAACTTTAAAAAAATA

TAGCATAAAAGTCAAATTATTAAAAACTTTTTCACTTGAAATTCTTTCAACTTTGACCAA

TTTAACTAGAAAGTAAATTTCATCTAAATTTTAACATTTTATATTAAAAACATGATACAA

CACTCAAAAGTATATTAACTACTTTGAGGAGAGTTTTAGAAATATATTTAATTTGGTAAC

ACAAAATTTGTCTGGAATATACTATTACCTTGAGAGTAATTAATACTGATTATAACAGAC

ACTCAATTAGCAAGCCTGCAGGAATTCGATTCTTTGAAAAGAGGCTTCATACTCCCTCCG

TTCATCTTCACTTGTTCATTATATTATTTAAGAATATTTAATAATATTTATCCAATTTAT

AAAATCAATGAATAATTTATCATTTTATTCTTGTTATTAAATATTATTTATTTTCCATAT

TCAGAATTATGGAGTATTAAATGAAATATTTAGTTCATGAATTAACTAGTACAAATTTTT

TTAAAATCACCTTATCGAGCATATTAAATGTAAATAATTAACGCAGCAAACTAACCTATG

AGATTTTCATATTCTCAGCTTTGCAAGCCGTTTGGAAAGACTTATTGGGGAACGTAGTCT

TACAAGTAACAGCAATAGTGCCGAGATCACCAAATGCATTGTGCTCAGTGTGAGAGGTTG

CACGAAGCTTCTTAGTCAAGCATTTCTCATTTGTATATCTTTTACGATTCTATCTATTAA

TGATTTTTGTATACATTTGATTGGCATATTTCTATATTTTCCAACCCATAGGTATTTGTA

TAATATTTTATATTTATGCAAATCATCATATAAGCAAGAAATTCGAATCAAAGAGCAAAT

ATATCAAAAATTTCAAAAAGATACCATACATGCACCACAAATAAGGTCTACTTTTTTCTA

TTGAAAATAAGATTTTTATTTTATTACTCCATTAATTAAAGATAAATAAACATAAAATAA

AACAAAGAGAAATATAAAAGTAATTTGTTTTTACAAATCACAAGCAATTAACTTGAAACA

GTAATCAAACTTTTTCTCTTATTATTAGTATTATAATACCAACAGGCATCAGAAGATTCT

CTCCAACACAGAATCATTTTCAAAGGCATCCCTGCAATTTATTAGACATGGAATATTTAG

TTGATGAACTAAATACAAATTTAAAAAAATCATGGATATTAAACGTAAATAATTATTTAT

AGTACCTGTGATATTCTCAACTTTGCAAGCCGTTCCGAGACCCTGAGACTAATTGGGGAA

ATGTTTGGGCCTTGTACAAGAAATAGCTTTTTCAATTTAGGCATACTCATATCATCCATG

CACGTTACTTCAGACAACCCTTGTGCATCCTCAATATACAAAACTTCAAGTTGCGGAAAG

CTATCACCAGAGAGATCCATTTTTTCTGCATCATGTCTGCACCAAAGCAATATAAGTATC

CTTAATTTGGGCAATTTCTTAAGCACTGCCAATAGATGACCGTCTACCATCAAGCCGACA

AGATTAAGCTTGACAAGATTTGGAGGGAATGCTTCAATGTTCAAGAGCATTGCTGAGAAA

CCAACCAAACCCAACTCGACAATATTTGGATGGGACGACAAGTTTATTTGCTCACTCGTG

TTCTTGAAAAATCTGAGCTTCAGAACCTCCAACGCTCTAGGCACAGGGCTCAATGCTGAT

AATATCTTAATGGTAGAACCGGATACATCCATTATACACAATGTTCTTAAATTTATCAAT

CGGTGCAACAATCTTGGTTCACAAAATTTATCATCCACCCACATCAAAGTTTGTAGATTA

TTAGGAGGCATGATTCGGCAAAATGGACTTGCAAAAGAGACACAGTAACATTCTTCTGTG

TAACAAAGATGTCTCAATATTTTAGACTCCCAAACACCAAAAGGAAGTTTACTAGAGCTA

TGAAATATATCCAGGGTCTCTAGACATTTGAGCTTGACAATACTATTTGGCAATTTTACT

CTAATTGCCCCCTCCAATCGCACATAACGTAGGCAACTCATGTTCCCAATTTTTTTGGGG

AAAGTAACATGCTGATAACCCTTTTGAGACATGACTACAACCAACACTTGCAATAATTTG

AAGTTAAGATGAGCCATTTGAGAAAATATGCAACGGTCTTTTGTGAAACAGAAAAGTGAA

CGAAGCTTCATAGGCTTAGGATTTAAATGGAAGAACTCATTCATGGCATTATCATCAGAG

TAGAATGTAATCCTTCGCACCCTAGCAACATTGCTAGGATCACCAAATGCATTGTGCTCC

GTGTGAAAGAAGTTACTTTCCTTAGCCAAGTCCACACACAAACTATGTAACAAGTCATGT

ATGCGACAACTTGAAATTCTTCCATCATATGTCCTTTTGGCAACTTGAATCAAGTTTCTT

GAAACCAAATCATTTAGGACATCATCCGCCAAACTTTCAGCCTCTCGCCCATTGCCAGTA

TTTACAACTATCAGCTTCTCAGCAATCCACATATTTGTCAAATCAAAAGCACGAATTTCA

TGGTCCTCGGGGTAAAGACCAAAGTACAAGAAACATGGCCTTAATGCAATGGGCAAATCA

TTGTAACTCAGAGCCAATACCTTACCACATCCATCTTGAATTTTATGAGCCATACTCTCA

AGTACTCTGTTCCATGCATGTTCTGTTCTTCCTCTTGCCCTTAACATGCCTGCAGTCACC

ACAATTGCTAGCGGTATACCTCCACATCTCTCAACTATACATCTACCAATATTTACCAAG

TCTGGTGAAGCATTGGCCCAATTATCATTAACAAAATTAAAGATTTTCTTGGTAAAGAGT

TCAAAGCTTTTCTCTGAATCTAGGGGTTGCAACACGTGGATTGAGAAATCCCCTCCTATG

TATCTGCCTACATTACTATTTCGAGAGGTTATAATTATCCTACTGCCAATTTTTGAATCA

CATTCAGGAAGGACAAGTTTTAGATCATCCCAAATTTCAACATCCCAAATGTCATCTAAG

AGAATAACATACCTTTTTATTTTCAAGAGTGATCGTAGGTTGTTCTCCAAGTTTTCTTTC

CTTTCCTCTTCCGTCAGTCCAACTTGTTTGGCTATGTCATGTAAGATTTCTCCCGCCCTT

GGCTGTTGTGAAACATAGACCAGTCCCGAACACTCAAATTGATGACAGACATGCCTATAA

AGTTTCTTGGCAAGAGTTGTTTTTCCCAAACCGGGCATGCCAACTATTGAAACAACTCCA

TAAGGCAAATCATGATCAAGTAATTTGGCTTGTAGTGTATTGAAGTCATCTTCCAGACCG

ATGACCTCTGTTTCATCAGCATGAAGGAACAATCTTCTCCGGTCCAATGGAATGCAATCA

TCATTATTGTTACTTGTATCTGTGATGCTGTAAGTTGTCCTTACACGGTCAATATCAGCA

ACTCTTCTTTTTATCTTCTCAATCTCCATAGCAAACTCATCGGCAAAAGAAACCGTCTTA

AGGCAACAAATGAACTTATTGGATTGTTGAATTTTTGGAAGAAACTCATCTAATAGATCC

TCCACATCACCTGCCAGTTGTTGAATATCTTTTAATAAGTTTTTCACCCTTGAATCGCCT

CCAACTTCCTTTGCCTTTGCATTGTCTACATATGATCGAATGTGTCTCATTTCTCTCTGG

AGCCAATCGATGTCCTCTTTCAACCAATATAAACGCGTACCTTCTTGAAAGAGTACATTT

CCAGCTATTTCTATTGATTTATTGATGACTGCTGTGAGAAGAATTTCAGCCATCTCCTTT

AATTTTTCGTTAAATTTCTTTTTTCTAAATGTGAGGATGAGGAAGGGAAAGTAAGAGTCA

ACGGCCCAAGTCTTGTAAACACAATAATTCATCTTTTGTTAGCTGGTATTTCGTTTTCAA

GCATGAATAACAAACGTTGAAAGTCAGAAAATTCAGGCAGTACTACGCGTAAAACGAAAT

TAGAAAGAAGAAGATAATTGGTAAAGGAAAAGAAAGGTCAGCTTAGAATAGTTGGAGTTC

AGGGCCAAGTCTCCTAATAATGTCAAGTAAATGAAAAACGAAAGAAGAAAGGAACAACAC

GTGCTGTGGATAGATACTAGTAGAATAGTCAAAAGAAATTACACCGTTCGTCCCAATTTA

AGTGTCATTCAATTAAATTCGACATGGAATTTAAAAAACAAACAGTAACTTTTAATATTT

TGTGACTGATGATTGATAATGTACCTTTTTTTTTTGAAAAAATTACGTAACTAAGCAAAC

ATCTTTAGTATATAACTATCAATCACACCATATGTTAAAAAAATTTATTATATATCTAGC

GCCATTTTATGTTTCCTAACTGTTTGATGAACGTCTTGCAAAACTTTACCCAATTTGGGT

TCATGGCTGTGAGTTTGGGCTATTATGAGACTCAATTTGTATGTTCCATGAAAATTAGGC

TTTTATGCAACTTGATTCTCTCAAAATCTTTTGCCATACTCATACAGAGTGGGCGTTCGG

TTCGATATTTATTTAACTTTGATTTGATTTGGTCGTGAGACATCATCATAATGACTCGAG

TAGAATGTAGGGTGTATAACTAATCACTAGTGGAATTCCTGCAGGATCCCCGGGTACCTG

ACAGGATATATTGGCGGGTAAAC
