# Supplementary material for: Development of late blight resistant potatoes by cisgene stacking
Source: BMC Biotechnol. 2014 May 29;14:50. doi: 10.1186/1472-6750-14-50 (PMC4075930; doi:10.1186/1472-6750-14-50)
Supplement: Additional file 4 — Primers used for PCR analysis of transformants. [file 1472-6750-14-50-S4.pptx]

## Slide 1
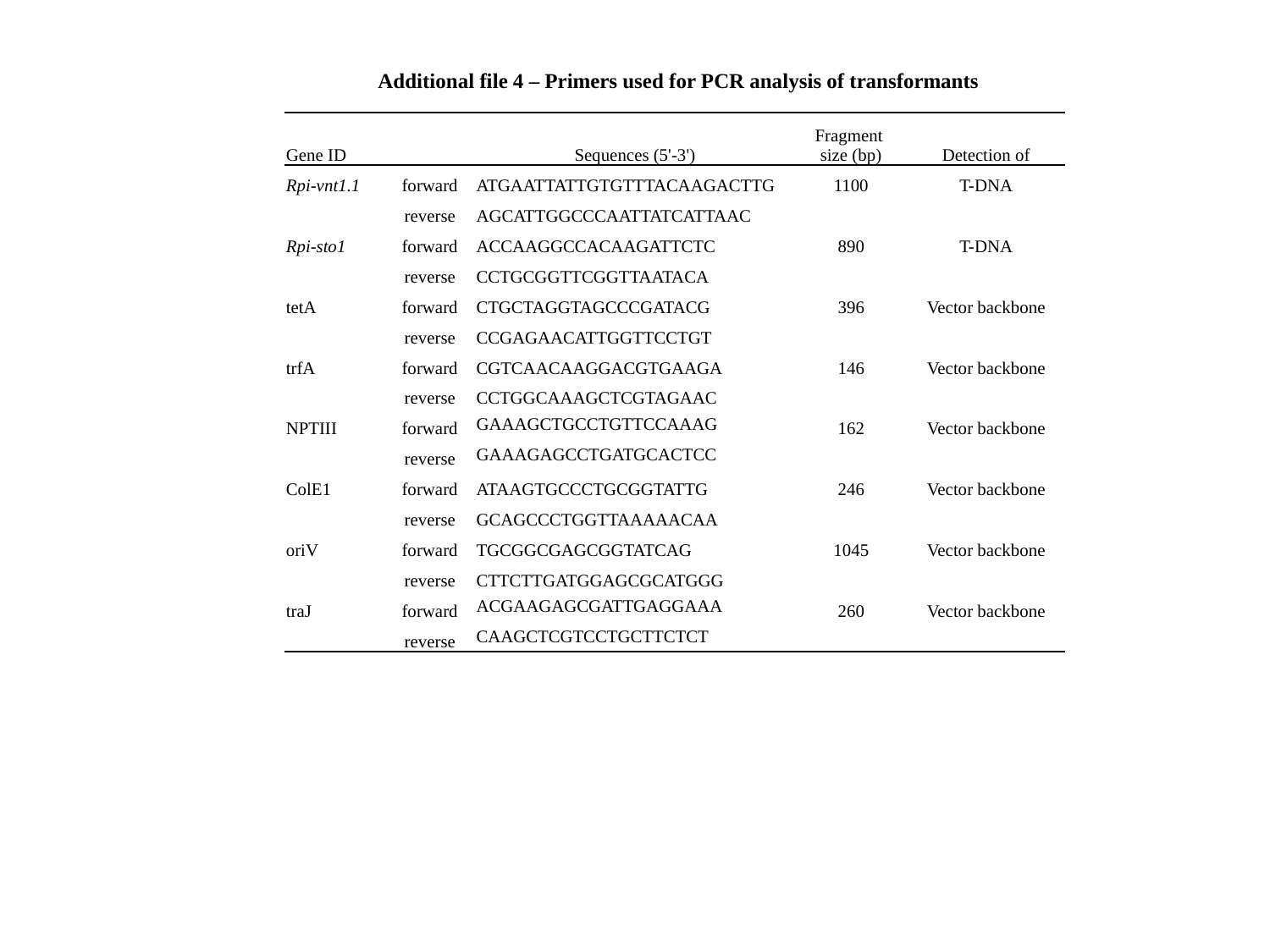

Additional file 4 – Primers used for PCR analysis of transformants
| Gene ID | | Sequences (5'-3') | Fragment size (bp) | Detection of |
| --- | --- | --- | --- | --- |
| Rpi-vnt1.1 | forward | ATGAATTATTGTGTTTACAAGACTTG | 1100 | T-DNA |
| | reverse | AGCATTGGCCCAATTATCATTAAC | | |
| Rpi-sto1 | forward | ACCAAGGCCACAAGATTCTC | 890 | T-DNA |
| | reverse | CCTGCGGTTCGGTTAATACA | | |
| tetA | forward | CTGCTAGGTAGCCCGATACG | 396 | Vector backbone |
| | reverse | CCGAGAACATTGGTTCCTGT | | |
| trfA | forward | CGTCAACAAGGACGTGAAGA | 146 | Vector backbone |
| | reverse | CCTGGCAAAGCTCGTAGAAC | | |
| NPTIII | forward | GAAAGCTGCCTGTTCCAAAG | 162 | Vector backbone |
| | reverse | GAAAGAGCCTGATGCACTCC | | |
| ColE1 | forward | ATAAGTGCCCTGCGGTATTG | 246 | Vector backbone |
| | reverse | GCAGCCCTGGTTAAAAACAA | | |
| oriV | forward | TGCGGCGAGCGGTATCAG | 1045 | Vector backbone |
| | reverse | CTTCTTGATGGAGCGCATGGG | | |
| traJ | forward | ACGAAGAGCGATTGAGGAAA | 260 | Vector backbone |
| | reverse | CAAGCTCGTCCTGCTTCTCT | | |
